# Polyphenols in Cocoa and Cocoa Products: Is There a Link between Antioxidant Properties and Health?

**DOI:** 10.3390/molecules13092190

**Published:** 2008-09-16

**Authors:** Abbe Maleyki Mhd Jalil, Amin Ismail

**Affiliations:** Department of Nutrition and Dietetics, Faculty of Medicine and Health Sciences, Universiti Putra Malaysia 43400, UPM Serdang, Selangor Darul Ehsan, Malaysia

**Keywords:** Cocoa, polyphenols, antioxidants, methylxanthines

## Abstract

Cocoa and cocoa products have received much attention due to their significant polyphenol contents. Cocoa and cocoa products, namely cocoa liquor, cocoa powder and chocolates (milk and dark chocolates) may present varied polyphenol contents and possess different levels of antioxidant potentials. For the past ten years, at least 28 human studies have been conducted utilizing one of these cocoa products. However, questions arise on which of these products would deliver the best polyphenol contents and antioxidant effects. Moreover, the presence of methylxanthines, peptides, and minerals could synergistically enhance or reduce antioxidant properties of cocoa and cocoa products. To a greater extent, cocoa beans from different countries of origins and the methods of preparation (primary and secondary) could also partially influence the antioxidant polyphenols of cocoa products. Hence, comprehensive studies on the aforementioned factors could provide the understanding of health-promoting activities of cocoa or cocoa products components.

## Introduction

The chronology of cocoa began in 2,000 B.C, the date attributed by historians to the oldest drinking cups and plates that have ever been discovered in Latin America at a small village in the Ulúa valley in Honduras, where cocoa played a central role. In 200-900 AD, cocoa was one of the main products in Mayan agriculture and religion. For instance, cocoa is used as a gift to deceased dignitaries at their funeral ceremonies and as currency [1]. The word cacao is derived from the Olmec and the subsequent Mayan languages (kakaw) and the chocolate-related term cacahuatl is Nahuatl (Aztec language) derived from Olmec/Mayan etymology [2]. In 1737, the cocoa tree was named *Theobroma cacao* which refers to the mythical background of the tree literally means “cocoa, food of the gods” [1]. Dillinger *et al*. [2] reported that medicinal uses of cocoa had been traced from Mexican (Aztec) sources and approximately 150 uses of cocoa for medical treatment had been documented. Various parts of *Theobroma cacao* have been utilized, namely cocoa beans prepared as chocolate, cocoa bark, cocoa butter, cocoa flower, cocoa pulp and cocoa leaf. Cocoa was brought to Europe by the Spanish in 1505. By 1653, cocoa was used in Europe as a medicine rather than as a delicious foodstuff. The use of chocolate was recognized as stimulating the healthy function of the spleen and other digestive functions. Moreover, in the 17th and 18th century, chocolate was regularly prescribed or mixed into medications for all sorts of ailments and diseases from colds and coughing, to promote digestion, fertility, reinforce mental performance and as an anti-depressant [1].

Studies on the health benefits of cocoa and cocoa products have been conducted over the past decade, with a major focus on degenerative diseases. These benefits could be due to their significant amounts of flavonoid monomers (catechin and epicatechin) up to tetradecamers [3,4]. It was noted that all polyphenols possessed antioxidant action *in vitro*, but do not necessarily exert antioxidant potential *in vivo* [5]. Most of the early studies focusing on health benefits of cocoa polyphenols came from human clinical trials [5]. Moreover, the study on health benefits of cocoa was not limited to that of human intervention but had also been extensively studied *in vitro* and *in vivo* [6,7,8,9,10]. 

The majority of the studies have examined the contributions of the flavonoids in cocoa and cocoa-products towards health benefits, but it must be noted that cocoa and their products are also rich in methylxanthines, namely caffeine, theobromine and theophylline [11,12] and studies have demonstrated that methylxanthines can possess both positive and negative health effects. For instance, caffeine intake has been reported to have negative effects on reproductive health [13]. On the other hand, caffeine supplementation enhanced net hepatic glucose uptake through increment of glucose-6-phosphate production in the liver [14]. Cocoa is also rich in micronutrients [15] and micronutrients such as copper found in cocoa could contribute significantly towards human dietary intake [16]. As cocoa contains a mixture of bioactive components, it is possible to postulate that there may be direct or indirect synergism between these components in delivering their health properties. Cooper *et al*. [5] suggested that if the biological effects are due to cocoa flavonoids rather than the other components, the perfect control would be cocoa or cocoa products that contain things other than flavonoids.

Factors affecting quality and quantity of cocoa and cocoa-based products during production and manufacturing are of great importance in delivering the best health effects. These factors could significantly reduce the polyphenol content of the selected products. Physiological factors, namely bioavailability and antioxidant properties, should also be considered when assessing their contribution in the biological system. For instance, there could be a significant correlation between interactions of polyphenols and proteins, but the interference of protein with polyphenols remains inconclusive, as some studies have demonstrated that protein could reduce the activity of polyphenols and some studies not [17,18]. To date, there is a lack of studies which concurrently determine the outcomes and the bioavailability or antioxidant status of the studied subjects. Studies reported that antioxidant status of the subjects are not improved or enhanced although there were positive health outcomes. Hence, is there actually a link between cocoa antioxidants and health or is it due to any other components of the cocoa and cocoa products? Factors such as bioavailability, types of cocoa and cocoa products used in the intervention study, antioxidant status, and the state of subjects being studied (normal, borderline, or with disease) would at least affect the measured outcomes.

## Polyphenols and other components in cocoa beans and cocoa-based products

### Polyphenols

Cocoa had long been identified as a polyphenols-rich food. The main polyphenol in cocoa or known as cacao was first identified by Ultée and van Dorsen in 1909 [19]. The crystalline compound they discovered, with empirical formula C_16_H_16_O_6_, was called “Kakaool”. For over 20 years afterwards, there was disagreement between researchers in naming this phenolic compound. Further purification indicated that this compound was catechin, with empirical formula C_15_H_14_O_6_ [20]. However, this compound was mistakenly named l-acacatechin as one of catechins present in the cutch-producing acacias (*Acacia catechu*). A year later, Freudenberg *et al*. [21] reported that “Kakaool” probably represented l-epicatechin, which could also be found in *Acacia catechu*. Later, they agreed that the name l-acacatechin was incorrect as both catechin and epicatechin are stereoisomers. Forsyth [22] reported that cocoa bean contains four types of catechins, of which (-)-epicatechin constitutes about 92%. 

Adam *et al*. [23] indicated that unfermented cocoa bean contains both tannin and catechin. The brown and purple color of the cocoa bean was attributed to the complex alteration products of catechin and tannin. Beside these compounds, cocoa was found to have leucoanthocyanins that are present as glycosides. Similarly, it was observed that cocoa beans contains two cyanidin glycosides and at least three leucocyanidins (procyanidin) compounds [22]. Leucocyanidins constitute about 60% of total polyphenols in fresh cocoa cotyledon [24]. Quesnel [25] found that cocoa bean contains simple dimeric leucocyanidin and epicatechin. Epicatechin and simple leucocyanidins 1, 2 and 3 (L1, L2, and L3) are present in cocoa beans, as identified using two-dimensional paper chromatography. In addition, cyanidin is also present as cyanidin-arabinoside and cyanidin-galactoside. Later in 1977, Jalal *et al*. [26] indicated that the major components of cocoa extracts (leaves, cotyledons, stem, and callus) are anthocyanins, leucocyanidins, (-)-epicatechin, catechin, p-coumaryl quinic acid and chlorogenic acid.

**Figure 1:**
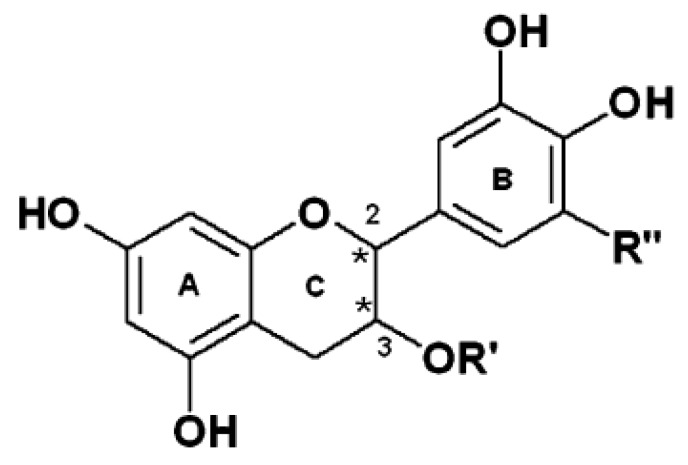
Basic structure of flavonols [34].

**Figure 2:**
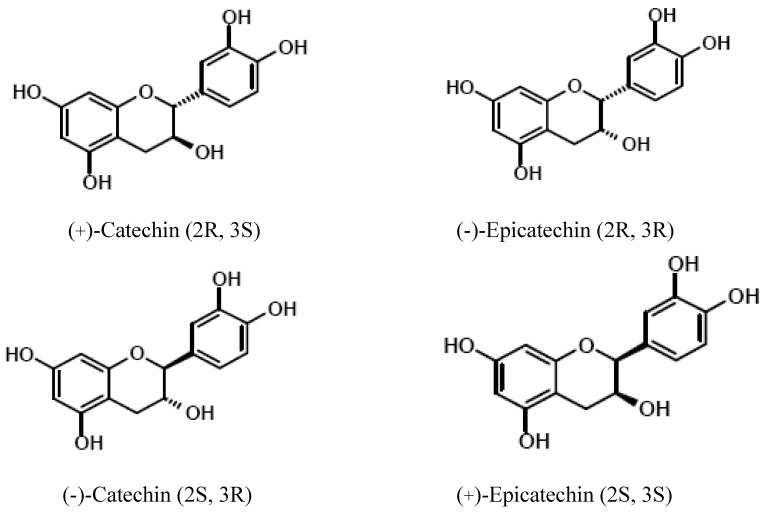
Structures the catechin and epicatechin enantiomers [34,46].

**Figure 3:**
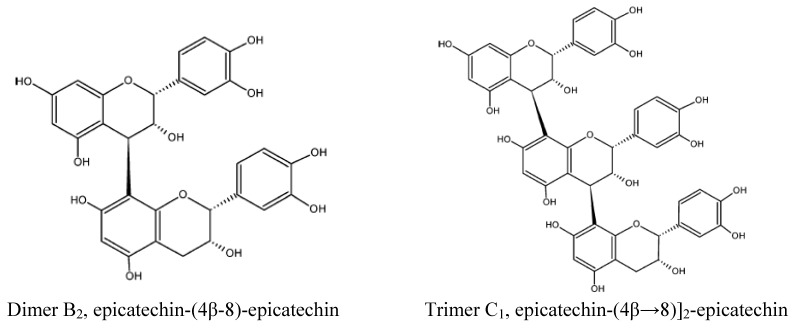
Structures of procyanidin dimer and trimer in cocoa [36].

The study on cocoa polyphenols became more extensive with the discovery of major low molecular weight polyphenols in cocoa, namely catechin, epicatechin, dimers epicatechin-(4β→8)-catechin (procyanidin B1), epicatechin-(4β→8)-epicatechin (procyanidin B2), and trimer [epicatechin-(4β→8)]_2_-epicatechin (procyanidin C1) [27]. Previous studies showed that monomeric polyphenols, namely epicatechin and catechin, dimer, trimer, and tetramer were detected by reverse-phase liquid chromatography mass spectometry (RP LC-MS) [28]. It has been reported that flavonols (epicatechin and catechin) were predominant compounds in cocoa powder [29,30]. Epicatechin was predominant in all chocolates, with a ratio of 1:0.1, compared to catechin [31]. The basic structure of flavanols is shown in Figure 1. Structures of the monomeric catechin and epicatechin enantiomers are shown in Figure 2, while the dimer and trimer are given in Figure 3. The monomers are stereoisomers at position 3 of the C-ring, but have the same configuration at position 2 [32]. Moreover, the interflavan bond at position 4 is always *trans* to the hydroxyl (OH) group at position 3. (+)-Catechin and (–)-epicatechin forms are commonly found in cocoa [33]. However, their respective enantiomers namely (–)-catechin and (+)-epicatechin are not commonly found in nature [34]. The chemical structures of flavonols and procyanidins are important for their antioxidant activity as they possess both free radical trapping and chelation of redox-active metals properties [35]. Flavonoids and procyanidins were found to prevent lipid oxidation through interaction between lipid forming membranes and the adsorption to the polar lipid headgroups [36]. 

* indicates chiral center located at the C_2_ and C_3_-position of the C-ring.

Porter *et al*. [37] confirmed that another three new compounds are present in fresh cocoa beans. The new compounds were identified as epicatechin-(2β→5,4β→6)-epicatechin, 3T-*O*-β-d-galacto-pyranosyl-ent-epicatechin-(2α→7,4α→8)-epicatechin and 3T-*O*-l-arabinopyranosyl-ent-epicatechin-(2α→7,4α →8)-epicatechin. Cocoa was reported to have high polyphenols content, which comprises 12-18% of the whole beans dry weight [38]. A study reported that in raw cocoa beans, 60% of total phenolics were flavonol monomers (epicatechin and catechin) and procyanidin oligomers [39]. (–)-Epicatechin content in freshly prepared beans ranged from 21.89-43.27 mg/g dry defatted samples [40]. Cocoa is rich in polyphenols such as (+)-catechin, (–)-epicatechin, and oligomers of these monomeric base units, namely procyanidins, and anthocyanidins [41]. Kelm *et al*. [3] later indicated that unfermented cocoa beans contain monomers up to tetradecamers.

Recently, it has been showed that chocolate is one of the most polyphenol-rich foods along with tea and wine [42,43]. Results indicated that dark chocolate exhibited the highest polyphenol content, with 610 mg total catechins/kg of fresh edible weight [43]. With advancements in technology, high performance liquid chromatography (HPLC) had been utilized in the determination of polyphenol compounds in cocoa. Kim and Keeney [44] developed a sensitive method for determination of (-)-epicatechin in cocoa beans which was detected at 280 nm and quantified by using external standards. Chocolates is also rich in polyphenol substances, such as (–)-epicatechin (EC), (+)-catechin, quercetin (including its glucoside), clovamide, deoxyclovamide, trans-resveratrol and its glucoside (trans-piceid) and procyanidin [41,45].

Over the past decade, at least 28 studies have been reported on the health benefits of cocoa flavonoids [5]. Most studies showed positive relationships between cocoa and chocolate flavonoids on cardioprotective effects. These findings were attributed to antioxidant flavonoids ranging from monomers to oligomers in cocoa and chocolates as discussed before. However, most of the outcomes were based on the short-term effects (between 4 days to 6 weeks) [5]. Is there a link between the antioxidant flavonoids of cocoa and cocoa products on health effects based on short-term studies? Hence, long-term feeding trials of cocoa and cocoa products on health benefits are warranted. As reported, bioavailability of ingested flavonoids present in cocoa or cocoa-based products is of great importance as it may in turn reflect antioxidant status of the studied subjects. Thus, it is important to consider both bioavailability and antioxidant status in determining the relationship between cocoa flavonoids and health benefits.

The measurement of plasma antioxidant concentration and oxidative stress levels are examples of determining antioxidant status. Effects of monomers up to decamers derived from cocoa was dose-dependent and prevented erytrocyte hemolysis *in vitro* and enhanced plasma antioxidant capacity [47]. Adamson *et al*. [48] indicated that polyphenol content positively correlated with antioxidant properties as measured by oxygen radical absorbance capacity (ORAC). All polyphenols possess antioxidant properties *in vitro* but are not likely to exert the same properties *in vivo* and in human. An *in vivo* study indicated that epicatechin from cocoa could enhance the antioxidative activity of plasma [49]. Physiologically, epicatechin exhibited dose-dependence in plasma after dark chocolate consumption as low as 1 nM [50]. The presence of epicatechin (12-fold from baseline) leads to significant increase in plasma total antioxidant capacity and decrease in plasma thiobarbituric acid reactive substances. Similarly, Wang *et al*. [51] demonstrated that dark chocolate dose-dependently increased plasma antioxidants and decrease 8-isoprostane. 

Cocoa supplementation exerts promising health properties due to its antioxidative properties, however, there are also studies which failed to show these effects. Supplementation of cocoa for four weeks significantly improves platelet function among healthy subjects [52]. However, there was no correlation between cocoa intake and plasma antioxidant status. Similarly, cocoa intake decreased LDL oxidation without changes in antioxidant potentials and oxidative stress level in plasma [53]. Dark chocolate supplementation for three weeks in healthy subjects significantly increased high-density lipoprotein cholesterol (HDL-c) compared to their unsupplemented counterparts [54]. However, there were no changes in total antioxidant capacity and oxidative stress biomarker (8-isoprostane). Similarly, Wan *et al*. [55] demonstrated that cocoa powder and dark chocolate supplementation improved HDL levels by 4% compared to control diet, but there were no changes in oxidative stress biomarkers. Milk chocolate bar consumption increased HDL levels compared to high-carbohydrate snacks among young men [56]. These studies clearly indicated that cocoa administration did not exert their antioxidative properties in plasma of healthy subjects, although there were significant health outcomes. This could be due to the status of subjects recruited in the study. Cooper *et al*. [5] reported that healthy subjects may already have optimum dietary status and supplementation will not produce meaningful outcomes. 

Numerous studies have been reported on the health benefits of cocoa and their products on cardiovascular diseases [5]. The measured outcomes were mainly focused on plasma antioxidant activity [51], low density lipoprotein (LDL) oxidation [57], blood pressure [58], arterial flow mediated dilation (FMD) [59] and platelet aggregation [52]. Most of the studied outcomes of cocoa polyphenols on cardiovascular health were on the antioxidant status, endothelial function, inflammatory production, nitric oxide bioactivity and platelet function [60]. These factors were associated with coronary diseases [61]. Most of the studies showed cocoa enhanced flow mediated dilation. Flow mediated dilation (FMD) of the brachial artery is a tool for measuring endothelium-dependent dilation [62]. The results indicated that a single dose of cocoa drink increased nitric oxide in human plasma and improved endothelial dysfunction. Moreover, Balzer *et al.* [63] reported that cocoa ingestion improved basal FMD by 30% without changes in endothelial function, blood pressure, heart rate and glycaemic control among diabetics. Procyanidins extracted from cocoa exhibited endothelium-dependent relaxation (EDR) through activation of nitric oxide synthase activity in rabbit aortic rings *in vitro* [64]. The results were reported to be due to the tetramers and higher polymers of epicatechin, and monomers, dimers, and trimers were not capable of contributing to EDR. *In vivo* studies indicated that dark chocolate, cocoa powder and cocoa liquor suppressed the development of atherosclerotic lesions and inhibited atherosclerosis [9,10]. High-flavonoid content dark chocolate (containing 259 mg polyphenols) significantly improved FMD in healthy subjects compared to low-flavonoid chocolate (containing trace amount of polyphenols) [65]. Although the supplementation of high-flavonoids dark chocolate increased plasma epicatechin, there were no significant changes in LDL oxidation, total antioxidant capacity, 8-isoprostane, blood pressure, body weight and body mass index. In contrast, there was a study which showed negative association between flavonoids-rich chocolate consumption and cardiovascular diseases among coronary artery disease (CAD) subjects [66]. There were no significant changes observed in lipid profiles, soluble cellular adhesion molecules, FMD, systemic arterial compliance and forearm blood flow. The negative outcomes could be due to several factors which include the age of subjects, and their CAD burden.

A growing number of studies were done on cocoa polyphenols and their protective effect towards LDL oxidation as an early indicator for the development of cardiovascular diseases [53,67,68]. Mathur *et al*. [53] demonstrated reduced LDL oxidation after cocoa supplementation for 6 weeks compared to unsupplemented subjects. Baba *et al*. [67] indicated that after 4 weeks of low, medium, and high dosages of cocoa powder supplementation, subjects showed decreased plasma LDL cholesterol, oxidized LDL and apo-B concentration compared to baseline. Long-term supplementation of cocoa powder (12-weeks) to normo and mildly hypercholesterolemic human subjects had decreased LDL oxidation and increased plasma HDL cholesterol compared to the control group [68]. In addition, cocoa powder supplementation in healthy males significantly prevented LDL oxidation [57]. 

Low doses of dark chocolate (containing 30 mg polyphenols) supplementation to prehypertension subjects for 18 weeks significantly reduced systolic and diastolic blood pressure compared to polyphenols-free white chocolate [58]. Dark chocolate consumption (containing 180 mg polyphenols) resulted in reduction of total and LDL cholesterol among elevated serum cholesterol subjects [69]. There was also significant reduction in systolic blood pressure.

Cocoa liquor showed dose-dependently prevents the development of hyperglycemia in diabetic obese mice [70]. To a greater extend, our previous studies indicated that 4 weeks of cocoa powder extract supplementation to diabetic animal model showed hypolipidemic and hypoglycaemic properties [71,72]. In human subjects with hypertension, dark chocolate administration ameliorated insulin [73,74]. Brand-Miller *et al*. [75] reported that incorporation of cocoa powder as flavour in six different foods (chocolate bars, cakes, breakfast cereals, ice creams, flavored milks and puddings) increased postprandial insulin secretion compared to strawberry flavour. However, no changes were observed in the glycaemic index. They suggested that specific insulinogenic amino acids may explain their findings. Unlike cardiovascular diseases, there is still limited human study on the effects of cocoa polyphenols and diabetes in human clinical trials. More work need to be done in exploring the effects of cocoa polyphenols on diabetes risk.

Cocoa powder exerted anti cancer properties in *in vivo* studies. Amin *et al*. [76] indicated that cocoa liquor extract lower the activity of tumor marker enzymes during hepatocarcinogenesis. Cocoa powder supplementation significantly reduces the incidence of prostate carcinogenesis compared to positive controls [77]. The supplementation also increased the life span of the tumor-bearing rats. Bisson *et al*. [78] reported that cocoa powder dose-dependently decreased prostate hyperplasia through reducing dihydrotestosterone level and prostate size ratio. To the greater extend, long-term supplementation of cocoa powder improve cognitive performance in aged rats compared to unsupplemented rats [79]. Daily cocoa extract administration prevented the overproduction of free radicals after heat exposure and thus protect from cognitive impairments [80].

### Methylxanthines

**Figure 4:**
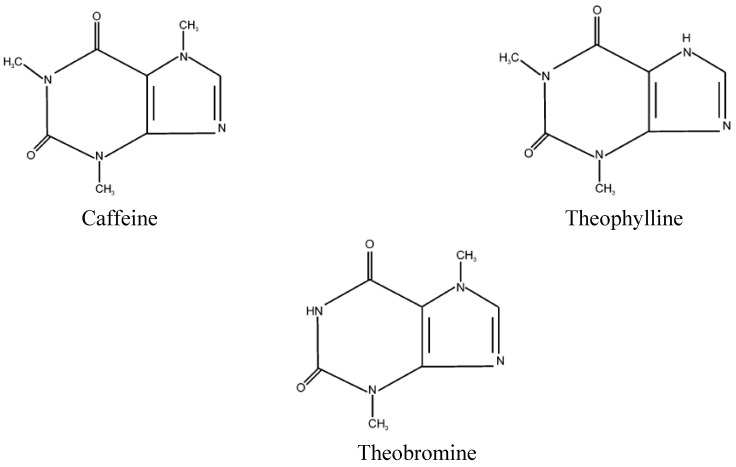
Methyxanthines present in cocoa [86].

Apart from polyphenols, cocoa is also rich in methylxanthines, namely caffeine, theobromine, and theophylline [11,12]. These compounds, found in dark chocolates, are responsible for chocolate cravings [81]. Caffeine was found in cocoa beans in 1909 [19]. It was initially found as a mixture of caffeine and catechin known as “caffeine-kakaool”. Forsyth indicated that caffeine can form a loose complex with epicatechin [82]. Later, Forsyth and Quesnel [24] indicated that only theobromine was identified in cocoa beans. Theobromine is the major methylxanthine present in cocoa, constituting about 4% on a fat free basis, while the caffeine content is about 0.2% [83]. Pura Naik [84] has confirmed that theobromine is the dominant purine alkaloid present in cocoa beans. In contrast, theophylline was present in low amounts [85]. The structures of methylxanthines present in cocoa are shown in Figure 4. Theobromine is also a major alkaloid in young pericarp and is present almost exclusively in the cotyledons of the beans. Caffeine and 3-methylxanthine are the major alkaloids in mature pericarp. 

Dark chocolate is high in theobromine and caffeine due to the addition of high amount of cocoa solids than that of milk and white chocolates. The amount of theobromine is higher, compared to caffeine in cocoa and cocoa products [11,12,58]. Theobromine is a psychoactive compound without diuretic effects. Caffeine levels are relatively low in cocoa, compared to those found in coffee and tea [15]. Although most of the studies indicated that the health benefits of cocoa or cocoa products were attributable to polyphenols [5], it should be noted that cocoa and cocoa products are not only rich in polyphenols, but are also rich in methylxanthines (caffeine, theobromine, and theophylline). Most of the studies underline the effects of polyphenols on the studied subjects, but the question of whether the presence of methylxanthines enhances or reduces the health benefits of the cocoa flavonoids remains unanswered, as the evidence in favor or against is often contradictory. Furthermore, the possible synergistic interactions between flavonoids and methylxanthines are also unclear and need further study. Kelly [87] has suggested that the contribution of theobromine in dark chocolate towards health benefits should be considered. For instance, methylxanthines particularly caffeine, could exert pro-oxidant properties, and caffeine, theobromine, and theophylline exerted antioxidant activity and protective ability under physiological conditions [88], but Vinson *et al*. [89] have reported that theobromine and caffeine were neither prooxidant nor antioxidant. To date, the studies on the effect of caffeine on glucose metabolism have also demonstrated conflicting outcomes. 

Administration of 5 mg caffeine/kg body weight reduced insulin-stimulated glucose uptake in T2DM and sedentary human subjects as measured by hyperinsulinemic-euglycemic clamp procedure [12,90]. Ingestion of 5 mg/kg body weight caffeinated coffee resulted in increased area under the curve for glucose, insulin, and C-peptide compared to decaffeinated coffee among healthy men [91]. In diabetics, caffeine supplementation had adverse effects on glucose metabolism and impaired postprandial glucose response [92]. Graham *et al*. [93] showed that the same dose of caffeine could also increase serum insulin and C peptide compared to placebo. Mechanistically, methylxanthines inhibited phosphodiesterase and hence increase the intracellular concentration of cyclic adenosine monophosphate. It was known that intracellular concentration of cyclic adenosine monophosphate is involved in the regulation of both insulin secretion from pancreatic cells and liver glucose output. The presence of methylxanthines may thus positively affect glucose metabolism [94]. 

Caffeine supplementation enhanced net hepatic glucose uptake through increment of glucose-6-phosphate production in the liver during glucose load [14]. Aminophylline (a type of caffeine metabolite) has been shown to stimulate glucose and arginine-stimulated insulin release [95,96]. Previously, it had been reported that adenosine receptor stimulates glycogenolysis in hepatocytes through binding to A_2_ receptor [97,98] and inhibit insulin secretion through binding to A_1_ receptor [99,100]. Arias *et al*. [101] indicated that aminophylline supplementation decreased glucose production with concomitant increased in insulin secretion, indicating aminophylline inhibits endogenous glucose production in type 2 diabetes. 

Eteng and Etarh [102] demonstrated that single doses of theobromine (700 mg/kg body weight) significantly reduced lipid profiles in hyperlipidemic rats. However, no effect on lipid profiles was observed after administration of cocoa extract, compared to pure theobromine. Eteng *et al*. [103] reported that supplementations of 3% and 15% cocoa powder that contained 56 to 265 mg theobromine to the rats significantly reduced body weight and also decreased lipid profiles. These results indicated that cocoa possessed hypocholesterolemic properties, in contrast to the first study. This is probably due to the dosage used as the theobromine content in the first study was almost three times higher than in the later one. It was noted that theobromine was responsible for the activation of hormone-sensitive lipase, which hydrolyzed triacylglycerols and release free fatty acids and glycerols from adipose tissue into plasma [104].

A study has demonstrated the positive effects of theobromine on cancer. Theobromine was reported to potentially inhibit angiogenesis induced by ovarian cancer cells through inhibition of vascular endothelial growth factor production [105]. At a therapeutic dose of 500 mg/kg it has been used in the treatment of cardiac oedema and angina pectoralis, and its analogues pentoxyfylline, suramin-theobromine and lisophylline are currently being exploited for cancer chemotherapy [106,107,108]. In contrast, theobromine intakes among men have been associated with increased risk of prostate cancer [109]. 

Previously, theobromine intakes had been reported to have negative effects on reproductive health. Theobromine (250 mg theobromine/kg body weight) can lead to vacuolation within the Sertoli cells, altered spermatid shape, and failure in the release of late spermatids in male rats [13]. Moreover, high-dose cocoa extract containing theobromine could alter testis structure to a greater extent to that of pure theobromine. The supplementation caused changes in weight patterns and in the morphology of the thymus in both sexes of rabbits. Theobromine supplementation causes mortality in rabbits. Soffietti [110] indicated that 1 and 1.5% of theobromine supplementation causes mortality in mature and immature rabbits and the effects were reported to be both dose- and time-dependent. Strachan and Bennett [111] did indicate sudden death of laboratory animals after theobromine administration, due to cardiac failure. In pregnant women, caffeine from coffee or cocoa beverages is freely absorbed through the placenta and eventually leads to fetal growth retardation [112,113]. 

### Peptides

Besides polyphenols and methylxanthines, cocoa is also rich in proteins. Cocoa peptides are generally responsible for the flavour precursor formation [114,115,116]. Cocoa beans contain four types of proteins, namely albumins, globulins, prolamin, and glutelin. Of these, albumin constitutes the major protein fraction [117,118]. Albumin and globulin fractions accounted for 52% and 43% of total bean proteins, respectively [119]. Albumin has a molecular weight of 19 kDa, while the globulins have molecular weights of 47, 31, and 14.5 kDa. Cocoa bean is the first to have vicilin-like globulin with sedimentation coefficient of 7-8S and a molecular weight of 150 kDa. The storage protein comprises two vicilin fractions with molecular weights of 47.1 and 39.2 kDa, and the albumin fraction has a molecular weight of 21.1 kDa [120]. Buyukpamukcu *et al*. [121] reported two new compounds were formed from vicilin protein during cocoa fermentation. The peptides are nonapeptide and hexapeptides (a product of nonapeptide degradation). 

To the best of our knowledge, there are few studies on the contribution of cocoa peptides towards health. Cocoa is also rich in peptides and amino acids as described in the previous section. It has been found that peptides and amino acids are responsible for the taste and aroma precursors of chocolate. Bioactive peptides were reported to possess antihypertensives, antithrombotic, hypocholesterolemic and hypotriglyceridemic, and antiobesity effects [122]. Marcuse [123] reported that the antioxidant activity was attributable to histidine, tyrosine, methionine and cysteine. Of these, histidine possessed strong radical scavenging activity due to the decomposition of its imidazole ring [124]. In addition, hydrophobicity of peptides also appears to be an important factor for their antioxidant activity, due to increased interaction with hydrophobic targets (e.g. fatty acids) [125]. However, the health effects of peptides in humans and the optimal plasma levels remain to be elucidated [122]. 

### Minerals

As discussed, the health properties of cocoa and cocoa products are not solely dependent on their polyphenol contents, but also contributed to by other components such as methylxanthines (caffeine and theobromine), peptides, and minerals. Previous studies seem to underestimate the contribution of minerals and peptides in cocoa and cocoa products towards health benefits. Steinberg *et al*. [126] showed that minerals are one of the important components in cocoa and cocoa products. Cocoa and cocoa products contained relatively higher amount of magnesium compared to black tea, red wine, and apples [15]. Based on Dietary Reference Intakes (DRI) of magnesium for men and women with an age range between 30-71 years old, cocoa powder provides 53% of DRI as per serving size (44 g) followed by dark chocolate (12%), sweet chocolate (12%), milk chocolate (7%), cocoa drink (1%), and white chocolate (1%). In human subjects with hypertension, dark chocolate administration ameliorated insulin [73,74]. The dose of dark chocolate (100 g) given contains 115 mg magnesium, which approximately delivers 36% of DRI per day. Hence, questions arise as to whether there is any direct contribution of magnesium besides polyphenols to health benefits? 

Magnesium is an important mineral in the regulation of blood pressure. It was observed that magnesium intake from foods inversely related to blood pressure compared to intake from supplements [127]. The highest quantile (median intake = 457 mg/day) of magnesium intake in Health Professionals Follow-up Study was associated with reduced risk of developing cardiovascular disease compared to lowest quantile (median = 269 mg/day) [128]. The amount of magnesium (440 mg) in this study is almost equivalent to magnesium content in 88 g unsweetened dry cocoa powder [15]. It is therefore possible that high magnesium content could partially contribute towards the health benefits of cocoa and cocoa-based products. Magnesium was also found to be positively associated with insulin sensitivity in Insulin Resistance Atherosclerosis Study [129]. Moreover, epidemiological study indicated that magnesium was inversely related to the development of metabolic syndrome [130,131]. A prospective study (European Prospective Investigation Into Cancer and Nutrition (EPIC)–Potsdam Study) indicated that magnesium may reduce diabetes risk [132]. Moreover, Kandeel *et al*. [133] showed that intracellular magnesium deficiency leads to the development of insulin resistance.

Cocoa and cocoa products are also rich in copper and can contribute significantly to daily copper intake. According to Dietary Reference Intakes, cocoa powder (44 g) provides 189% of daily copper intake followed by dark chocolate (34%), sweet chocolate (28%), milk chocolate (24%), white chocolate (3%) and cocoa drinks (2%). Hence, copper could be considered as one of the health contributors present in cocoa and cocoa products. Superoxide dismutase is a metalloenzyme (copper may be located in the center of the enzyme structure) that is involved in the dismutation of the superoxide anion to molecular oxygen and hydrogen peroxide [134,135]. Joo *et al*. [136] indicated that copper in cocoa and chocolate had significantly contributed to human diet. Dark chocolate is the largest contributor of copper intakes in the US followed by chocolate milk, chocolate syrup, milk chocolate and chocolate cake. Moreover, a positive association was found between total copper intakes and consumption of chocolate [136]. 

According to the USDA database [15], cocoa powder (44 g) could provide 11% selenium followed by milk and white chocolate (4%), dark chocolate (3%), sweet chocolate (2%), and cocoa drink (1%). Selenium is an essential micronutrient as cofactor in the formation of glutathione peroxidases, thioredoxin reductase, iodothyronine deiodinases, selenophosphate synthetase, selenoprotein P and other selenoproteins [137]. Specifically, it works by detoxification of hydrogen peroxide and organic peroxides [135]. A study reported that low level of selenium is directly related to diabetic nephropathy [138]. Selenium exhibited antioxidant properties by fully or partially restoring antioxidant enzymes in rat tissue [139]. Moreover, selenium exerted antioxidant ability through free radical scavenging and inhibition of lipid peroxidation [140]. Based on the significant contribution of selenium to antioxidant enzyme, it is important to consider the contribution of selenium in cocoa and cocoa products. 

## Factors affecting the quantity and quality of polyphenols in cocoa beans and cocoa-based products

Recently, various types of dark chocolates are available in the market with high flavonoid contents. These chocolates are produced by controlling bean selection, fermentation, and reduced heat and alkalization treatments [141]. Furthermore, there are chocolates producers who produced chocolates from high-flavonoids beans from Ecuador and utilized special roasting methods that preserve flavonoids in the cocoa beans [141]. By controlling the process involved in preparing the chocolates, a high-flavonoids chocolate can be produced that preserves up to 70% of the flavonoids present in the finished product. Most of the intervention studies used dark chocolate. This is due to the fact that dark chocolate contains more non-fat cocoa solid (cocoa powder) than other chocolates. The quantity of the flavonoids present in these products solely depends on the amount of non-fat cocoa solid content. On the other hand, white chocolate is prepared with cocoa butter and sugar without cocoa powder. Thus, dark and milk chocolates are expected to have flavonoids, while white chocolate will have none. Thus, the selection of the best quality cocoa and cocoa products could deliver the best antioxidant flavonoids. 

### Countries of origin

Cocoa from different varieties exhibited differences in polyphenols content by up to 4-fold [142]. Moreover, cocoa beans from different origins contain different amount of (–)-epicatechin and (+)-catechin. Cocoa beans from Ecuador possessed the highest amounts of (–)-epicatechin and (+)-catechin, followed by beans from Ghana and Trinidad [143]. Azizah *et al*. [144] also reported that cocoa beans from different countries may have different polyphenols content. They found that the highest phenolic content was in Malaysian beans followed by Sulawesian, Ghanian and Côte d’Ivore.There was about 6-fold variation in epicatechin contents in fermented cocoa beans from different regions [40].

Cocoa beans undergo various stages of processing before turning into raw cocoa (fermented and dried cocoa beans) and cocoa-based products. Cocoa products are products that were prepared from cocoa or cocoa-related components namely cocoa liquor, cocoa butter, and the products ranging from chocolates, cakes and pastries, mousses and crème, and drinks. Krawczyk [141] showed that flavonoid content of cocoa products depends on the cocoa beans used to make them, for example type of cocoa bean, origins, and amount added in the production of the products. It is important to know the factors which influence the polyphenols content of finished products. 

### Fermentation

Fermentation is one of the steps involved in the production of cocoa beans. This step is crucial in determining the quality of cocoa aroma. The production of aroma precursors during fermentation is important for producing the full aroma of chocolate [145,146]. There are internal and external fermentation stages involved during cocoa fermentation. External fermentation primarily involves the catabolism of the sugar pulp by microorganisms, while internal fermentation encompasses the biochemical changes in the cotyledon of the beans [145,147]. 

Research has shown that chocolates produced from unfermented beans have no chocolate flavor and are excessively astringent and bitter [148]. Fermentation will reduce the level of bitterness and astringency of the cocoa bean which could be attributed to the loss of polyphenols during fermentation [40,142,149]. Oxidation of polyphenols to insoluble tannins during fermentation was responsible for the formation of flavour precursors for chocolate processing [150,151,152]. Catechin has a bitter taste with a sweet aftertaste or is described as bitter and astringent [153]. Stark *et al*. [154] showed that catechins, which include epicatechin, catechin, procyanidin B2, procyanidin B5 and procyanidin C1, were the major compounds responsible for bitterness and astringency of roasted cocoa. However, Stark *et al*. [154] reported that the bitter taste and astringency were not just attributable to polyphenols, but were also contributed to by amino acids.

The aforementioned factors are directly or indirectly related to the quantity and quality of polyphenols content of cocoa and cocoa products. Moreover, the level of flavonoids is also dependent on the processing steps with focus on the fermentation. Fermentation is considered as one of largest influences on flavonoid levels in chocolates [141]. The conversion of simple cyanidin compounds to more complex leucocyanidin is the main change in the polyphenolic compounds in cocoa cotyledons. The change has of great importance in determining the flavour of the final product [82]. Pettipher [155] demonstrated that procyanidins are converted to largely insoluble red-brown material resulting in the characteristic colour of chocolate during fermentation and roasting. To date, numerous studies have been reported on the loss of compounds in cocoa beans during these processes. According to Wollgast and Anklam [156] fermentation is an essential stage that lasts from five to six days in the development of suitable flavour precursors.

During fermentation, polyphenols diffuse from their storage cells and undergo oxidation to become condensed high molecular compounds mostly insoluble tannins. This process involves both non-enzymatic and enzymatic, and catalyzed by polyphenol oxidase. Even though this enzyme is strongly inactivated during the first days of fermentation, about 50 and 60% of enzyme activity after first and second days is still remained, respectively [157]. Furthermore, approximately 10 to 20% of epicatechin and other soluble polyphenols are reduced during fermentation. This could also due to the diffusion of polyphenols into fermentation sweating [40,157,158]. Caligiani *et al*. [143] reported that (–)-epicatechin and (+)-catechin increased in the order of fully fermented (brown color), partly fermented (violet color) and unfermented (slaty color). In addition, high temperatures and prolonged processing times will decrease the amount of catechins [156]. During fermentation, between days two and three, epicatechin content was observed to decrease sharply, which could indicate that it is either being used up for the formation of large tannins or lost in the fluids that drain away [40].

Different degrees of roasting significantly increased the amount of (+)-catechin due to the isomerization of (–)-epicatechin. Kyi *et al*. [159] demonstrated that the high temperature used during drying of fermented cocoa beans had reduced polyphenol contents as a result of enzymatic oxidation. Non-enzymatic oxidation of polyphenols could also occur at this stage. Almost 90% of polyphenols are lost from fresh cocoa beans during fermentation. Polyphenols content gradually decreased upon fermentation from days 0 to 8 [153]. Tomas-Barberan *et al*. [4] quantified the content of dimers and trimers in unfermented and unroasted cocoa powder. Dimers are present in cocoa powder produced from fermented and dried cocoa beans. As cocoa powder is derived from fermented, dried, and roasted cocoa beans, the loss of phenolic compounds is higher than that of cocoa liquor [160]. Furthermore several phenolic compounds were undetected in cocoa powder produced from fermented, dried, and roasted beans compared to cocoa powder produced from unfermented beans [4]. In practice, cocoa manufacturers would blend the unfermented, partly fermented and fully fermented beans, to obtain the desired flavour characteristics and also to reduce the excessive astringency and bitterness. The bitterness of the chocolates was also due to the presence of flavonoids. Thus, manufacturers tend to remove them in large quantities to enhance taste quality. Apart from this, the manufacturers tend to prefer Ghanian cocoa beans, which are well-fermented and flavorful than that of Dominican or Indonesian beans, which are considered as less fermented and have low quality cocoa flavor [161]. Therefore, the flavonoid content may not be detected in cocoa beans. 

The introduction of heating during manufacturing of chocolates and cocoa-based products can change the enantiomeric composition of (+)-catechin [162]. In addition, the epimerization of catechins could be caused by the conditions applied during the extraction procedures. It has been shown that two days of sun-drying of fresh unfermented cocoa beans (without fermentation) causes a 50% decrease in epicatechin content. This process may reduce the epicatechin content gradually during the process of making chocolates starting from fresh cocoa beans. However, most of the beans used for chocolates manufacturing are fermented, yet, it is far from being a standardized process throughout the world, or even within a region. 

### Manufacturing process

Chocolate is made from different recipes and contains other ingredients in addition to cocoa butter and powder [163]. The basic ingredients required for the manufacturing of chocolate are cocoa liquor, sugar, other sweeteners, cocoa butter, oil, milk powder, milk crumb and emulsifiers. Chocolates may have different percentages of non-fat cocoa solids. There are different percentages of cocoa butter, sugar, and milk powder are used in making different types of chocolates namely dark chocolate, milk chocolate, and white chocolate. The content of polyphenols can vary tremendously depending on the source of beans, primary and secondary processing conditions, and process of chocolates making. Due to these factors, the ratio and types of polyphenols found in cocoa beans are unlikely to be the same as those found in the finished products [31]. Alkalization (or dutching) of cocoa powder will reduce the polyphenol content and antioxidant activity [48,164]. 

According to Cooper *et al*. [5], the presence of non-fat cocoa solid (NFCS) is an excellent marker to determine the total phenolic content. Miller *et al*. [161] showed that the highest NFCS was in cocoa powder (72-87%), followed by baking chocolate (45-49%), dark chocolate (20-30%), semi sweet chocolate (15-19%), milk chocolate (5-7%), and chocolate syrup (5-7%). Dark chocolates contain the highest NFCS among the different types of chocolates. Theoretically, the higher amount of non-fat cocoa solid indicates the higher phenolic content in the chocolates. Furthermore, there was positive and significant relationship between NFCS and antioxidant properties [161]. Vinson *et al*. [89] reported that the amount of polyphenols increased in the order of hot chocolate < milk chocolate < dark chocolate < cocoa. In addition, Cooper *et al*. [165] reported that the percentage of cocoa which appears on chocolates' labeling cannot be used accurately to estimate the polyphenol contents, since it includes polyphenol-free cocoa butter. This may overestimate the polyphenols content in certain type of chocolates. Furthermore, cocoa polyphenols contain different structures of flavonoids, thus creating difficulties in the determination of polyphenols content in chocolates. 

Epicatechin has a strong correlation with other polyphenols compared to catechin [32]. The relationships suggest that polyphenols are affected to the same degree. A study showed that fresh cocoa beans contain (+)-catechin with undetectable levels of the (–) form [162]. The presence of the (–)-catechin in chocolates could be due to epimerization of the (–)-epicatechin during the manufacturing of chocolates. Epicatechin has been reported as the main polyphenols present in cocoa beans [40,166]. Usually, it does not correlate well with the cocoa and polyphenols content, especially the chocolates that are high in total polyphenols, such as dark chocolates [165]. This may follow the agreement that epicatechin is well established as the main polyphenols found in cocoa beans. A recent study showed that roasted cocoa beans and cocoa products contained flavan-3-ol (–)-catechin along with (+)-catechin and (–)-epicatechin. The compound was formed during manufacturing process through epimerization of (–)-epicatechin and its epimer (–)-catechin [46]. As reported, typical RP-HPLC analysis was limited in separating enantiomers of catechin and epicatechin.

### Bioavailability of cocoa polyphenols

Recently, there have been two major approaches commonly used to determine the availability of phenolic compounds either by measuring their concentration in plasma and urine after ingestion of known amount of foodstuffs or ingestion of the pure compounds [167]. The bioavailability of polyphenols greatly depends on the chemical structure, glycosylation, acylation, conjugation, and polymerization. Different forms of catechins either (+) or (–) are absorbed differently. According to Donovan *et al*. [168], the concentration of (–)-catechin was higher to that of (+)-catechin in chocolates. However, bioavailability of (–)-catechin was less than the (+) form of catechin, resulting in low plasma concentration of (–)-catechin. On the other hand, Fraga *et al*. [169] reported that (+)-catechin was 100 times more efficient than quercetin in an *in vivo* oxidative stress model. 

Monomeric flavonoids are absorbed in the small intestine, but polymeric procyanidins may be degraded by intestinal and colonic microflora followed either by absorption of the metabolites or excretion in the feces. After absorption, the monomers and dimers may be methylated, sulfated, or glucuronidated in the liver [167]. Besides physiological factors, food matrix and texture, the presence of other nutrients (protein, carbohydrate, and fat) and the interaction between them may directly or indirectly affect the bioavailability of polyphenols. In a human clinical trial, the administration of 148 mg of procyanidins had increased plasma epicatechin at 2 h compared to baseline (0 h) [170]. In addition, plasma epicatechin concentration increased to 21.2 nmol/L after consumption of the procyanidins and then returned to normal levels [170]. Similarly, plasma procyanidin dimer, (–)-epicatechin and (+)-catechin can be detected as early as 0.5 h and reach maximal concentrations by 2 hr after acute consumption of cocoa [171]. Murphy *et al*. [172] indicated that administration of procyanidins increased plasma (–)-epicatechin and (+)-catechin by 81% and 28%, respectively. Moreover, (–)-epicatechin was detected as early as 0.5 to 1 hr after chocolate or cocoa consumption and they are present mainly as sulfate conjugated, glucuronides, or methylated forms [173]. A study related to plasma kinetics of epicatechin in men after consumption of 40 g and 80 g of dark chocolates, detected that epicatechin increased markedly after chocolates consumption, reaching a maximum between 2-3 hours [174]. The maximal concentration and area under the curve of plasma kinetics correlate well with the dose of chocolates. This indicates that epicatechin is absorbed from chocolates and is rapidly eliminated from plasma. Attainable plasma levels were 0.7 μmol/L (free epicatechin and epicatechin conjugates) from 80 g of black chocolates which contain 164 mg of epicatechin. To a greater extend, Baba *et al*. [175] indicated that administration of procyanidins B_2_ [epicatechin-(4β-8)-epicatechin] extracted from cocoa powder was absorbed in the plasma and excreted in the urine. The compounds appeared maximally in the plasma at 30 min and decreased gradually from 30 min to 300 min. 

Different types of polyphenols, polyphenol structures (+ or –), glycosylation, acylation, conjugation, and glycosides all showed different degrees of absorption. With the exception of catechin and epicatechin, most flavonoids are present as glycosides (attached to a sugar moiety). The absorption of these glycosides is dependent on the position of sugar linkage [176]. Glycosidic flavonoids require hydrolysis by colonic microflora to cleave the sugar and release the aglycone for absorption, whereas aglycones (without sugar moiety) can passively diffuse through the small intestine. As reported by Spencer *et al*. [177] only small amounts of procyanidins B2 and B5 were transferred to the serosal side of enterocytes in isolated small intestine. However, the transferred dimers resulted in unmetabolized/unconjugated epicatechin monomer. Baba *et al*. [173] reported that (–)-epicatechin from chocolate or cocoa are present in plasma of human volunteers as sulfate, glucuronide, and sulfate-glucuronide (mixture of sulfate and glucuronide) conjugates, rather than methylated forms. It was reported that plasma concentration of glucuronide conjugates of non-methylated and methylated (–)-epicatechin were higher compared to other forms. Using a pure (–)-epicatechin compound, Da Silva [178] showed that (–)-epicatechin is present in plasma in the forms of glucuronide and sulfate-glucuronide conjugates (free and O-methylated) forms. The glucuronidation of (–)-epicatechin occurs at the position 3‘ of the B ring in humans [179]. Natsume *et al*. [179] found that (–)-epicatechin metabolites present in human plasma are different than the forms present in rats. The glucuronidation of (–)-epicatechin occurs at the 7 position of the A ring and the 3’ position of the B ring (Figure 1) in rats and humans, respectively. This glucuronidation compound showed low antioxidant activity compared to intact compounds as it has lost the catechol structure of the B ring responsible for the antioxidative effects. (–)-Epicatechin metabolites are present in urine within 24 hr in the range of 25% to 30%. There is a significant reduction in total (–)-epicatechin metabolites after 6 hr and the remaining conjugates are mostly present as the O-methylated form. Excretion of (–)-epicatechin metabolites in urine was observed to be dose-dependent [180]. Moreover, it was observed that the level of (–)-epicatechin metabolites excreted in urine was in the close range after equivalent ingestion of pure (–)-epicatechin and (–)-epicatechin from cocoa powder. Hence, the bioavailability of (–)-epicatechin was not influenced with the presence of other compounds present in cocoa powder. However, protein is one of the food factors that was widely studied due to their interaction with polyphenols. The presence of protein in food matrix may form complexes with polyphenols [181]. Serafini *et al*. [182] demonstrated that the presence of milk inhibits polyphenol absorption. Protein mainly from milk (chocolates) and digestive environment (salivary protein) may form complexes with polyphenols and reduce their bioavailability [18]. In addition, antioxidant capacity of polyphenols was modified with the presence of protein [183]. To date, the evidence for the interference of protein with polyphenols remains questionable. For instance, a study indicated that the addition of milk reduces the antioxidant capacity by 30% [18]. Schroeter *et al*. [17] however found that bioavailability of polyphenol monomer (epicatechin) was not reduced when cocoa is ingested with milk. It has been demonstrated that polyphenols bind to salivary protein and resulted in precipitation of insoluble complex and astringent flavour [184]. Moreover, high molecular weight polyphenols may form strong interaction with protein. Polyphenols not only bind to salivary protein but also with dietary protein and digestive enzymes in which in turn may influence their transportation and absorption activities [185]. The presence of protein either in foods matrix or in the digestive system could at least affect polyphenols bioavailability. 

Studies by Zhu *et al*. [186] and Klimczak *et al*. [187] reported that the degradation of catechin, epicatechin, and their dimers was influenced by physiological pH. Zhu *et al*. [188] indicated that auto-oxidation and epimerization were two major reactions involved in determining the stability of phenolic compounds under typical experimental conditions. Moreover, Rios *et al*. [33] reported that procyanidins were remarkedly stable in the stomach environment, and thus available for absorption or metabolism. Deprez *et al*. [189] showed that polymeric proanthocyanidins were degraded into low molecular weight aromatic compounds, namely, 2-(*p*-hydroxyphenyl)acetic acid, 2-(*p*-hydroxyphenyl) propionic acid, 2-(*m*-hydroxyphenyl)acetic acid, 2-(*m*-hydroxyphenyl)propionic acid, 5-(*m*-hydroxy-phenyl)valeric acid, and phenylpropionic acid after 48 h incubation with human colonic microflora. Similarly, flavanol-rich chocolate intake increased urinary excretion of phenolic acids, namely *m*‑hydroxyphenylpropionic acid, ferrulic acid, 3,4-dihydroxyphenylacetic acid, *m*-hydroxyphenylacetic acid, vanillic acid, and *m*-hydroxybenzoic acid [33]. Hence, it is clear that under the acidic environment in gastric milieu, procyanidins oligomers are hydrolyzed to monomeric epicatechin and dimer. The presence of these monomer and dimer could in turn enhance their absorption in the small intestine [190]. However, very little study has been done to investigate the effects of food components in our diet on the catabolism products of unabsorbed polyphenols in the guts because most of the high molecular weight polyphenols are not absorbed in the small intestine. In addition, most of the results were based on the normal and healthy subjects where the rate or extend of absorption are assumed to be normal. Relatively little studies have been reported on the bioavailability of flavonoids in subjects with disease. For example, there is a difference in the metabolism of polyphenols between normal and disease-state subjects. The concentration of naringenin metabolites was higher in healthy (17.3 μM) compared to tumor-bearing (10.6 μM) rats [191]. However, the nature of metabolites remains the same in plasma, tissues, and urine between the groups. To date, there is limited study on the bioavailability of cocoa flavonoids bioavailability in disease-state subjects. 

Unlike polyphenols, little is known of the presence of methylxanthines and their metabolites in human plasma and urine. Most of the studies reported the presence of polyphenols monomers (catechin and epicatechin) in plasma but not methylxanthines. Caffeine, theobromine, theophylline, and paraxanthine are four types of methylxanthines detected after 2-hr of cocoa intakes [192]. Of these, paraxanthine accounted for 67% of plasma methylxanthines followed by theobromine (24%), and theophylline (8%) [193]. However, plasma half-lives of theobromine and theophylline are 6.2 and 7.2 hr, respectively compared to caffeine and paraxanthine (4.1 and 3.1 hr, respectively) [194]. As cocoa contained significant amount of methylxanthines, it is interesting to know their synergistic effects with cocoa flavonoids. 

## Conclusions

There is a link between cocoa antioxidant and health due to the significant flavonoids content. However, the presence of methylxanthines, peptides and micronutrients could enhance or reduce the observed health effects. Factors such as bioavailability, antioxidant status, and state of subjects being studied may directly or indirectly affect the health benefits of cocoa polyphenols and the other components. This review opens new frontier on the health benefits of methylxanthines, peptides, and micronutrients in cocoa and cocoa-based products. Health benefits of these components could be explored in short- and long-term studies and among healthy and disease-state subjects.

## References

[1] (2008). Barry Callebaut. History of chocolates.

[2] Dillinger T.L., Barriga P., Escarcega S., Jimenez M., Lowe D.S., Grivetti L.E. (2000). Food of the Gods: Cure for humanity? A cultural history of the medicinal and ritual use of chocolate. J. Nutr..

[3] Kelm M.A., Johnson J.C., Robbins R.J., Hammerstone J.F., Schmitz H.H. (2006). High-performance liquid chromatography separation and purification of cacao (*Theobroma cacao* L.) procyanidins according to degree of polymerization using a diol stationary phase. J. Agric. Food Chem..

[4] Tomas-Barberan F.A., Cienfuegos-Jovellanos E., Marin A., Muguerza B., Gil Izquierdo A., Cerdaa B., Zafrilla P., Morillas J., Mulero J., Ibarra A., Pasamar M., Ramoan D., Espin J.C. (2007). A new process to develop a cocoa powder with higher flavonoid monomer content and enhanced bioavailability in healthy humans. J. Agric. Food Chem..

[5] Cooper K.A., Donovan J.L., Waterhouse A.L., Williamson G. (2008). Cocoa and health: a decade of research. Bri. J. Nutr..

[6] Ramljak D., Romanczyk L.J., Metheny-Barlow L.J., Thompson N., Knezevic V., Galperin M., Ramesh A., Dickson R.B. (2005). Pentameric procyanidin from *Theobroma cacao* selectively inhibits growth of human breast cancer cells. Mol. Cancer Ther..

[7] Ramiro E., Franch A., Castellote C., Andres-Lacueva C. (2005). Effect of *Theobroma cacao* flavonoids on immune activation of a lymphoid cell line. Bri. J. Nutr..

[8] Matsui N., Ito R., Nishimura E., Yoshikawa M. (2005). Ingested cocoa can prevent high-fat diet-induced obesity by regulating the expression of genes for fatty acid metabolism. Nutrition.

[9] Kurosawa T., Itoh F., Nozaki A., Nakano Y. (2005). Suppressive effects of cacao liquor polyphenols (CLP) on LDL oxidation and the development of atherosclerosis in Kurosawa and Kusanagi-hypercholesterolemic rabbits. Atherosclerosis.

[10] Vinson J.A., Proch J., Bose P., Muchler S., Taffera P., Shuta D., Samman N., Agbor G.A. (2006). Chocolate is a powerful *ex vivo* and *in vivo* antioxidant, an antiatherosclerotic agent in an animal model, and a significant contributor to antioxidants in the European and American Diets. J. Agric. Food Chem..

[11] Rios L.Y., Gonthier M.P., Remesy C., Mila I., Lapierre C., Lazarus S.A., Williamson G., Scalbert A. (2003). Chocolate intake increases urinary excretion of polyphenol-derived phenolic acids in healthy human subjects. Am. J. Clin. Nutr..

[12] Greer F., Hudson R., Ross R., Graham T. (2001). Caffeine ingestion decrease glucose disposal during a hyperinsulinemic-euglycemic clamp in sedentary humans. Diabetes.

[13] Wang Y., Waller D.P., Amiya P., Hikim S., Russell L.D. (1992). Reproductive toxicity of theobromine and cocoa extract in male rats. Reprod. Toxicol..

[14] Pencek R.R., Battram D., Shearer J., James F.D., Lacy D.B., Jabbour K., Williams P.E., Graham T.E., Wasserman D.H. (2004). Portal vein caffeine infusion enhances net hepatic glucose uptake during a glucose load in conscious dogs. J. Nutr..

[15] USDA (2008). United States Department of Agriculture, Nutrient Data Laboratory. http://www.nal.usda.gov/fnic/foodcomp/search/.

[16] Joo S., Kies C., Schnepf M. (1995). Chocolate and chocolate-like products: impact on copper status of humans. J. Appl. Nutr..

[17] Schroeter H., Holt R.R., Orozco T.J., Schmitz H.H., Keen C.L. (2003). Nutrition: milk and absorption of dietary flavanols. Nature.

[18] Tabernero M., Serrano J, Saura-Calixto F. (2006). The antioxidant capacity of cocoa products: contribution to the Spanish diet. Int. J Food Sci. Tech..

[19] Ultee A.J., van Dorsen J. (1909). Bijdrage tot de kennis der op Java gecultiveerde cacaosooten. Java Agric Station Report.

[20] Adam W.B., Hardy F., Nierenstein M. (1931). The catechin of the cocoa bean. J. Am. Chem. Soc..

[21] Freudenberg K., Cox R.F.B., Braun E. (1932). The catechin of the cacao bean. J. Am. Chem. Soc..

[22] Forsyth W.G.C. (1955). Cacao polyphenolic substances. III. Separation and estimation on paper chromatograms. Biochem. J..

[23] Adam W.B. (1928). Determination of the color-producing constituents of the cacao bean. The Analyst.

[24] Forsyth W.G.C., Quesnel V.C. (1963). The mechanism of cacao curing. Adv. Enzymol..

[25] Quesnel V.C. (1968). Fractionation and properties of the polymeric of the polymeric leucocyanidin of the seeds of *Theobroma cacao*. Phytochemistry.

[26] Jalal M.A.F., Collin H.A. (1977). Polyphenols of mature plant, seedling, and tissue cultures of *Theobroma cacao*. Phytochemistry.

[27] Thompson R.S., Jacques D., Haslam E., Tanner R.J.N. (1972). Plant proanthocyanidins. Part I. Introduction; the isolation, structure, and distribution in nature of plant procyanidins. J. Chem Soc [Perkin 1].

[28] Natsume M., Osakabe N., Yamagishi M., Takizawa T., Nakamura T., Miyatake H., Hatano T., Yoshida T. (2000). Analyses of polyphenols in cacao liquor, cocoa, and chocolate by normal-phase and reversed-phase HPLC. Biosci. Biotechnol. Biochem..

[29] Natsume M., Osakabe N., Takizawa T., Nakamura T., Miyatake H., Hatano T., Yoshida T., Ho. C-T., Zeng Q.Y. (2002). Analysis of polyphenol constituents in cocoa and chocolate. Quality Management of Nutraceuticals.

[30] Abbe Maleyki M.J., Amin I. Antioxidant properties of cocoa powder. J. Food Biochem..

[31] Cooper K.A., Campos-Gimenez E., Alvarez D.J., Nagy K., Donovan J.L., Williamson G. (2007). Rapid reverse-phase ultra-performance liquid chromatography analysis of the major cocoa polyphenols and inter-relationship of their concentration in chocolate. J. Agric. Food Chem..

[32] Stafford H.A., Lester H.H. (1980). Procyanidins (condensed tannins) in green cell suspension cultures of Douglas Fir compared with those in strawberry and avocado leaves by means of C8-reversed-phase chromatography. Plant Physiol..

[33] Rios L.Y., Bennett R.N., Lazarus S.A., Remesy C., Scalbert A., Williamson G. (2002). Cocoa procyanidins are stable during gastric transit in humans. Am. J. Clin. Nutr..

[34] Kofink M., Papagiannopoulos M., Galensa R. (2007). Enantioseparation of catechin and epicatechin in plant food by chiral capillary electrophoresis. Eur. Food Res. Technol..

[35] Verstraeten S.V., Oteiza P.I., Fraga C.G. (2004). Membrane effects of cocoa procyanidins in liposomes and Jurkat T cells. Biol. Res..

[36] Verstraeten S.V., Hammerstone J.F., Keen C.L., Fraga C.G., Oteiza P.I. (2005). Antioxidant and membrane effects of procyanidin dimers and trimers isolated from peanut and cocoa. J. Agric. Food Chem..

[37] Porter L.J., Ma Z., Chan B.G. (1991). Cacao procyanidins: major flavanoids and identification of some minor metabolites. Phyrochemistry.

[38] Richelle M., Tavazzi I., Offord E. (2001). Comparison of antioxidant activity of commonly consumed polyphenolic beverages (coffee, cocoa, and tea) prepared per cup serving. J. Agric. Food Chem..

[39] Dreosti I.E. (2000). Antioxidant polyphenols in tea, cocoa, and wine. Nutrition.

[40] Kim H., Keeney P.G. (1984). (-)-epicatechin content in fermented and unfermented cocoa beans. J. Food Sci..

[41] Hammerstone J.F., Lazarus S.A., Mitchell A.E., Rucker R., Schmitz H.H. (1999). Identification of procyanidins in cocoa (*Theobroma cacao*) and chocolate using high-perfomance liquid chromatography/ mass spectrometry. J. Agric. Food Chem..

[42] Arts I.C.W., Hollman P.C.H., Kromhout D. (1999). Chocolate as a source of tea flavonoids. The Lancet..

[43] Arts I.C.W., van de Putte B., Hollman P.C.H. (2000). Catechin contents of foods commonly consumed in the Netherlands. 1. Fruits, vegetables, staple foods, and processed foods. J. Agric. Food Chem..

[44] Kim H., Keeney P.G. (1983). Methods of analysis for (-)-epicatechin in cocoa beans by high-performance liquid chromatography. J. Food Sci..

[45] Sanbongi C., Osakabe N., Natsume M., Takizawa T., Gomi S., Osawa T. (1998). Antioxidative polyphenols isolated from *Theobroma Cacao*. J. Agric. Food Chem..

[46] Kofink M., Papagiannopoulos M., Galensa R. (2007). (-)-catechin in cocoa and chocolate: occurence and analysis of an atypical flavan-3-ol enantiomer. Molecules.

[47] Zhu Q.Y., Holt R.R., Lazarus S.A., Orozco T.J., Keen C.L. (2002). Inhibitory effects of cocoa flavanols and procyanidin oligomers on free radical-induced erythrocyte hemolysis. Exp. Biol. Med..

[48] Adamson G.E., Lazarus S.A., Mitchell A.E., Prior R.L., Cao G., Jacobs P.H., Kremers B.G., Hammerstone J.F., Rucker R.B., Ritter K.A., Schmitz H.H. (1999). HPLC method for the quantification of procyanidins in cocoa and chocolate samples and correlation to total antioxidant capacity. J. Agric. Food Chem..

[49] Baba S., Osakabe N., Natsume M., Yasuda A., Takizawa T., Nakamura T., Terao J. (2000). Cocoa powder enhances the level of antioxidative activity in rat plasma. Bri. J. Nutr..

[50] Rein D., Lotito S., Holt R.R., Keen C.L., Schmitz H.H., Fraga C.G. (2000). Epicatechin in human plasma: *in vivo* determination and effect of chocolate consumption on plasma oxidation status. J. Nutr..

[51] Wang J.F., Schramm D.D., Holt R.R., Ensunsa J.L., Fraga C.G., Schmitz H.H., Keen C.L. (2000). A dose-response effect from chocolate consumption on plasma epicatechin and oxidative damage. J. Nutr..

[52] Murphy K.J., Chronopoulos A.K., Singh I., Francis M.A., Moriarty H., Pike M.J., Turner A.H., Mann N.J., Sinclair A.J. (2003). Dietary flavanols and procyanidin oligomers from cocoa (*Theobroma cacao*) inhibit platelet function. Am. J. Clin Nutr..

[53] Mathur S., Devaraj S., Grundy S.M., Jialal I. (2002). Cocoa products decrease low density lipoprotein oxidative susceptibility but do not affect biomarkers of inflammation in humans. J. Nutr..

[54] Mursu J., Voutilainen S., Nurmi T., Rissanez T.H., Virtanen J.K., Kaikkonen J., Nyyssonen K., Salonen J.T. (2004). Dark chocolate consumption increases HDL cholesterol concentration and chocolate fatty acids may inhibit lipid peroxidation in healthy humans. Free Rad. Biol. Med..

[55] Ying Wan, Joe A Vinson, Terry D Etherton, John Proch, Sheryl A Lazarus, Penny M Kris-Etherton (2001). Effects of cocoa powder and dark chocolate on LDL oxidative susceptibility and prostaglandin concentrations in humans. Am. J. Clin. Nutr..

[56] Kris-Etherton P.M., Derr J.A., Mustad V.A., Seligson F.H., Pearson T.A. (1994). Effects of a milk chocolate bar per day substituted for a high-carbohydrate snack in young men on an NCEP/AHA Step 1 Diet. Am. J. Clin. Nutr..

[57] Osakabe N., Baba S., Yasuda A., Iwamoto T., Kamiyama M., Tokunaga T., Kondo K. (2004). Dose-response study of daily cocoa intake on the oxidative susceptibility of low-density-lipoprotein. J.Health Sci..

[58] Taubert D., Roesen R., Lehmann C., Jung N., Schomig E. (2007). Effects of low habitual cocoa intake on blood pressure and bioactive nitric oxide. JAMA..

[59] Heiss C., Kleinbongard P., Dejam A., Perre S., Schroeter H., Sies H., Kelm M. (2005). Acute consumption of flavanol-rich cocoa and the reversal of endothelial dysfunction in smokers. J. Am. Coll. Cardiol..

[60] Keen C.L., Holt R.R., Polagruto J.A., Wang J.F., Schmitz H.H. (2002). Cocoa flavanols and cardiovascular health. Phytochemistry Reviews.

[61] Engler M.B., Engler M.M. (2004). The vasculoprotective effects of flavonoid-rich cocoa and chocolate. Nutr. Res..

[62] Heiss C., Dejam A., Kleinbongard P., Schewe T., Sies H., Kelm M. (2003). Vascular effects of cocoa rich in flavan-3-ols. JAMA.

[63] Balzer J., Rassaf T., Heiss C., Kleinbongard P., Lauer T., Merx M., Heussen N., Gross H.B., Keen C.L., Schroeter H., Kelm M. (2008). Sustained benefits in vascular function through flavanol-containing cocoa in medicated diabetic patients. J. Am. Coll. Cardiol..

[64] Karim M., McCormick K., Kappagoda C.T. (2000). Effects of cocoa extracts on endothelium-dependent relaxation. J. Nutr..

[65] Engler M.B., Engler M.M., Chen C.Y., Malloy M.J., Browne A., Chiu E.Y., Kwak H-K., Milbury P., Paul S.M., Blumberg J., Mietus-Snyder M.L. (2004). Flavonoid-rich dark chocolate improves endothelial function and increases plasma epicatechin concentrations in healthy adults. J. Am. Coll. Nutr..

[66] Farouque H.M.O., Leung M., Hope S.A., Baldi M., Schechter C., Cameron J.D., Meredith I.T. (2006). Acute and chronic effects of flavanol-rich cocoa on vascular function in subjects with coronary artery disease: a randomized double-blind placebo-controlled study. Clin. Sci..

[67] Baba S., Natsume M., Yasuda A., Nakamura Y., Tamura T., Osakabe N., Kanegae M., Kondo K. (2007). Plasma LDL and HDL cholesterol and oxidized LDL concentrations are altered in normo and hypercholesterolemic humans after intake of different levels of cocoa powder. J. Nutr..

[68] Baba S., Osakabe N., Kato Y., Natsume M., Yasuda A., Kido T., Fukuda K., Muto Y., Kondo K. (2007). Continuous intake of polyphenolic compounds containing cocoa powder reduces LDL oxidative susceptibility and has beneficial effects on plasma HDL-cholesterol concentrations in humans. Am. J. Clin. Nutr..

[69] Allen R.R., Carson L.A., Kwik-Uribe C., Evans E.M., Erdman J.W. (2008). Daily consumption of a dark chocolate containing flavanols and added sterol esters affects cardiovascular risk factors in a normotensive population with elevated cholesterol. J. Nutr..

[70] Tomaru M., Takano H., Osakabe N., Yasuda A., Inouse K-I., Yanigisawa R., Ohwatari T., Uematsu H. (2007). Dietary supplementation with cacao liquor proanthocyanidins prevents elevation of blood glucose levels in diabetic obese mice. Nutrition.

[71] Ruzaidi A.M.M, Amin I., Nawalyah A.G., Hamid M., Faizul H.A. (2005). The effect of Malaysian cocoa extract on glucose levels and lipid profiles in diabetic rats. J. Ethnopharmacol..

[72] Ruzaidi A.M.M, Abbe M.M.J, Amin I., Nawalyah A.G., Muhajir H. (2008). Protective effect of polyphenol-rich extract prepared from Malaysian cocoa (*Theobroma cacao*) on glucose levels and lipid profiles in streptozotocin-induced diabetic rats. J. Sci. Food Agric..

[73] Grassi D., Lippi C., Necozione S., Desideri G., Ferri C. (2005). Short-term administration of dark chocolate is followed by a significant increase in insulin sensitivity and a decrease in blood pressure in healthy persons. Am. J. Clin. Nutr..

[74] Grassi D., Necozione S., Lippi C., Croce G., Valeri L., Pasqualetti P., Desideri G., Blumberg J.B., Ferri C. (2005). Cocoa reduces blood pressure and insulin resistance and improves endothelium-dependent vasodilation in hypertensives. Hypertension.

[75] Brand-Miller J., Holt S.H.A., de Jong V., Petocz P. (2003). Cocoa powder increases postprandial insulinemia in lean young adults. J. Nutr..

[76] Amin I., Koh B.K., Asmah. R. (2004). Effect of cacao liquor extract on tumor marker enzymes during chemical hepatocarcinogenesis in rats. J. Med. Food.

[77] Bisson J.-F., Guardia-Llorens M.-A., Hidalgo S., Rozan P., Messaoudi M. (2008). Protective effect of Acticoa powder, a cocoa polyphenolic extract, on prostate carcinogenesis in Wistar-Unilever rats. Eur. J. Can. Prev..

[78] Bisson J.-F., Hidalgo S., Rozan P., Messaoudi M. (2007). Therapeutic effect of ACTICOA powder, a cocoa polyphenolic extract, on experimentally induced prostate hyperplasia in Wistar-Unilever rats. J. Med. Food.

[79] Bisson J.-F., Nejdi A., Rozan P., Hidalgo S., Lalonde R., Messaoudi M. (2008). Effects of long-term administration of a cocoa polyphenolic extract (Acticoa powder) on cognitive performances in aged rats. Bri. J. Nutr..

[80] Rozan P., Hidalgo S., Nejdi A., Bisson J.-F., Lalonde R., Messaoudi M. (2006). Preventive antioxidant effects of cocoa polyphenolic extract on free radical production and cognitive performances after heat exposure in Wistar rats. J. Food Sci..

[81] Smit H.J, Blackburn R. J. (2005). Reinforcing effects of caffeine and theobromine as found in chocolate. Psychopharmacology.

[82] Forsyth W.G.C. (1952). Cacao polyphenolic substances. II. Changes during fermentation. Biochem. J..

[83] Timbie D.J., Sechrist L., Kenney P.G. (1978). Application of HPLC to the study of variables affecting theobromine and caffeine concentrations in cocoa beans. J. Food Sci..

[84] Pura Naik J. (2001). Improved high-performance liquid chromatography method to determine theobromine and caffeine in cocoa and cocoa products. J. Agric. Food Chem..

[85] Franzke C., Grunet K.S., Griehl H. (1969). Uber die bestimmung und den gehalt von theobromin und theophyllin in Mate, Kola und Kakao. Z. Lebensm. Unters.Forsch..

[86] Thomas J.B., James J.H., Schantz M.M., Porter B.J., Sharpless K.E. (2004). Determination of caffeine, theobromine, and theophylline in standard reference material 2384, baking chocolate, using reversed-phase liquid chromatography. J. Agric. Food Chem..

[87] Kelly C.J. (2005). Effects of theobromine should be considered in future studies. Am. J. Clin. Nutr..

[88] Lee C. (2000). Antioxidant ability of caffeine and its metabolites based on the study of oxygen radical absorbing capacity and inhibition of LDL peroxidation. Clin. Chim. Acta..

[89] Vinson J.A., Proch J., Zubik L. (1999). Phenol antioxidant quantity and quality in foods: cocoa, dark chocolate, and milk chocolate. J. Agric. Food Chem..

[90] Lee S., Hudson R., Kilpatrick K., Graham T.E., Ross R. (2005). Caffeine ingestion is associated with reductions in glucose uptake independent of obesity and Type 2 Diabetes before and after exercise training. Diabetes Care.

[91] Moisey L.L., Kacker S., Bickerton A.C., Robinson L.E., Graham T.E. (2008). Caffeinated coffee consumption impairs blood glucose homeostasis in response to high and low glycemic index meals in healthy men. Am. J. Clin. Nutr..

[92] Lane J.D., Feinglos M.N., Surwit R.S. (2008). Caffeine increases ambulatory glucose and postprandial responses in coffee drinkers with type 2 diabetes. Diabetes Care.

[93] Graham T.E., Sathasivam P., Rowland M., Marko N., Greer F., Battram D. (2001). Caffeine ingestion elevates plasma insulin response in humans during an oral glucose tolerance test. Can. J. Physiol. Pharmacol..

[94] Cerasi E., Luft R. (1969). The effect of an adenosine-3'5'-monophosphate diesterase inhibitor (aminophylline) on the insulin response to glucose infusion in prediabetic and diabetic subjects. Horm. Metab. Res..

[95] Cerasi E., Luft R. (1969). The effect of an adenosine-3'5'-monophosphate diesterase inhibitor (aminophylline) on the insulin response to glucose infusion in prediabetic and diabetic subjects. Horm. Metab. Res..

[96] Pontiroli A.E., Caviezel F., Alberetto M. (1992). Secondary failure of oral hypoglycaemic agents in lean patients with type 2 diabetes mellitus: Insulin sensitivity, insulin response to different stimuli, and the role of cyclic-AMP. Diabetes Metab..

[97] Hoffer L.J., Lowenstein J.M. (1986). Effects of adenosine and adenosine analogues on glycogen metabolism in isolated rat hepatocytes. Biochem. Pharmacol..

[98] Oetjen E.C., Schweickhardt K., Unthan-Fechner K. (1990). Stimulation of glucose production from glycogen by glucagon, noradrenaline and non-degradable adenosine analogues is counteracted by adenosine and ATP in cultured rat hepatocytes. Biochem J..

[99] Bertrand G., Petit P., Bozem M. (1989). Membrane and intracellular effects of adenosine in mouse pancreatic beta-cells. Am. J. Physiol..

[100] Hillaire-Buys D., Chapal J., Bertrand G. (1994). Purinergic receptors on insulin secreting cells. Fundam. Clin. Pharmacol..

[101] Arias A.M.P., Bisschop P.H., Ackermans M.T., Nijpels G., Endert E., Romijn J.A., Sauerwein H.P. (2001). Aminophylline stimulates insulin secretion in patients with type 2 diabetes mellitus. Metabolism.

[102] Eteng M.U., Ettarh R.R. (2000). Comparative effects of theobromine and cocoa extract on lipid profiles in rats. Nutr. Res..

[103] Eteng M.U., Ibekwe H.A., Umoh U.I., Ebong P.E., Umoh I.B., Eyong E.U. (2006). Theobromine rich cocoa powder induces weight loss and changes in lipid profile of obese Wistar rats. Discov. Innov..

[104] Granner D.K., Murray RK, Meyers PA, Granner DK, Rodwell VW (1990). Hormone action. Harper's Biochemistry.

[105] Barcz E., Sommer E., Sokolnicka I., Gawrychowski K., Roszkowska-Purska K., Janik P., Skopinska-Roozewska E. (1998). The influence of theobromine on angiogenic activity and proangiogenic cytokines production of human ovarian cancer cells. Oncol.Rep..

[106] Chang C.C., Chang T.C., Kao S.C., Kuo Y.F., Chien C.F. (1993). Pentoxyfylline inhibits the proliferation and glycosaminoglycan synthesis of cultured fibroblasts derived from patients with Grave's ophthalmopathy and pretibial myoedema. Acta Endocrinol. Copehn..

[107] Gil M., Skopinska R.E., Radomska D., Demkon U., Skurzak H., Rochowska M., Beauth J., Roszkowski K. (1993). Effect of purinergic receptor antagonists, suramin and theobromine on tumour-induced angiogenesis in BALB/C mice. Folia. Biol. Praha..

[108] Clark E., Rice G.C., Weeks R.S., Jenkins N., Nelson R., Bianco J.A., Singer J.W. (1996). Lisofylline inhibits transforming growth factor beta release and enhances trilineage hematopoietic recovery after 5- fluorouracil. Cancer Res. J..

[109] Slattery M.L., West D.W. (1993). Smoking, alcohol, coffee, tea, caffeine, and theobromine: risk of prostate cancer in Utah (United States). Cancer Causes Control.

[110] Soffietti M.G., Nebbia C., Valenza F., Amedeo S., Re G. (1989). Effects of theobromine in mature and immature rabbits. J. Comp. Pathol..

[111] Strachan T.R., Bennett A. (1994). Theobromine poisoning in dogs. Vet. Rec..

[112] Kimmel C.A., Kimmel G.L., White C., Grasto T.E., Young J.F., Nelson G.J. (1984). Blood flow changes and conceptual development in pregnant rats in response to caffeine. Fund. Applied Toxicol..

[113] Abdi F.B., Pollard I., Wilkinson J.M. (1993). Placental transfer and foetal disposition of caffeine and its metabolites in 20 day pregnant rat: A function of dose. Xenobiotica.

[114] Biehl B., Wewetzer C., Passern D. (1982). Vacuolar (storage) proteins of cocoa seeds and their degradation during germination and fermentation. J. Sci. Food Agric..

[115] Voigt J., Biehl B., Heinrichs H., Kamaruddin S., Marsoner G.G., Hugi A. (1994). *In-vitro* formation of cocoaspecific aroma precursors: aroma related peptides generated from cocoa-seed protein by co-operation of an aspartic endoprotease and a carboxypeptidase. Food Chem..

[116] Voigt J., Heinrichs H., Voigt G., Biehl B. (1994). Cocoa specific aroma precursors are generated by proteolytic digestion of the vicilin-like globulin of cocoa seeds. Food Chem..

[117] Zak D.K., Keeney P.G. (1976). Extraction and fractionation of cocoa proteins as applied to several varieties of cocoa beans. J. Agric. Food Chem..

[118] Zak D.K., Keeney P.G. (1976). Changes in cocoa proteins during ripening of fruit, fermentation, and further processing of cocoa beans. J. Agric. Food Chem..

[119] Voigt J., Biehl B., Syed Kamaruddin S.W. (1993). The major seed proteins of *Theobroma cacao* L.. Food Chem..

[120] Amin I., Jinap S., Jamilah B. (1997). Vicilin-class globulins and their degradation during cocoa fermentation. Food Chem..

[121] Buyukpamukcu E., David Goodall M., Hansen C-E., Keely B.J., Kochhar S., Wille H. (2001). Characterization of peptides formed during fermentation of cocoa bean. J. Agric. Food Chem..

[122] Erdmann K., Cheung B.W.Y., Schroder H. The possible roles of food-derived bioactive peptides in reducing the risk of cardiovascular disease. J. Nutr. Biochem..

[123] Marcuse R. (1960). Antioxidative effect of amino acids. Nature.

[124] Yong S.H., Karel M. (1978). Reaction of histidine with methyl linoleate: characterization of the histidine degradation product. J. Am. Oil Chem. Soc..

[125] Chen H.M., Muramoto K., Yamauchi F., Fujimoto K., Nokihara K. (1998). Antioxidative properties of histidine-containing peptides designed from peptide fragments found in the digests of a soybean protein. J. Agric. Food Chem..

[126] Steinberg F.M., Bearden M.M., Keen C.L. (2003). Cocoa and chocolate flavonoids: Implications for cardiovascular health. J. Am. Diet Assoc..

[127] Champagne C.M. (2008). Magnesium in hypertension, cardiovascular disease, metabolic syndrome, and other conditions: A Review. Nutr. Clin. Prac..

[128] Al-Delaimy W.K., Rimm E.B., Willett W.C., Stampfer M.J., Hu F.B. (2004). Magnesium intake and risk of coronary heart disease among men. J. Am. Coll. Nutr..

[129] Ma B., Lawson A.B., Liese A.D., Bell R.A., Mayer-Davis E.J. (2006). Dairy, magnesium, and calcium intake in relation to insulin sensitivity: approaches to modeling a dose-dependent association. Am. J. Epidemiol..

[130] He K., Liu K., Daviglus M.L., Morris S.J., Loria C.M., Van Horn L., Jacobs D.R., Savage P.J. (2006). Magnesium intake and incidence of metabolic syndrome among young adults. Circulation.

[131] Song Y., Ridker P.M., Manson J.E., Cook N.R., Buring J.E., Liu S. (2005). Magnesium intake, C-reactive protein, and the prevalence of metabolic syndrome in middle-aged and older U.S. women. Diabetes Care.

[132] Schulze Matthias B., Schulz M., Heidemann C., Schienkiewitz A., Hoffmann K., Boeing H. (2007). Fiber and magnesium intake and incidence of type 2 diabetes: a prospective study and meta-analysis. Arch. Intern. Med..

[133] Kandeel F.R., Balon E., Scott S., Nadler J.L. (1996). Magnesium deficiency and glucose metabolism in rat adipocytes. Metabolism.

[134] Colak E., Dimitrijevic-Sreckovic V., Djordjevic P.B., Stankovic S., Glisic B., Sreckovic B., Majkic-Singh N. (2008). Biomarkers of enzymatic and non-enzymatic antioxidative defense in type 2 diabetes mellitus–comparative analysis. Biochem. Med..

[135] Malstrom B., Andreasson L., Reinhammer B., Boyer P (1975). The Enzymes, XIIB.

[136] Joo S-J., Betts N.M. (1996). Copper intakes and consumption patterns of chocolate foods as sources of copper for individuals in the 1987-1988 nationwide food consumption survey. Nutr. Res..

[137] Letavayova L., Vlckova V., Brozmanova J. (2006). Selenium: from cancer prevention to DNA damage. Toxicology.

[138] Kornhauser C., Garcia-Ramirez J.R., Wrobel K., Perez-Luque E.L., Garay-Sevilla M.E., Wrobel K. (2008). Serum selenium and glutathione peroxidase concentrations in type 2 diabetes mellitus patients. Prim. Care Diabetes.

[139] Talas Z.S., Ozdemir I., Yilmaz I., Gok Y., Orun I. (2008). The investigation of the antioxidative properties of the novel synthetic organoselenium compounds in some rat tissues. Exp. Biol. Med..

[140] Luz Mora M.D.L., Pinilla L., Rosas A., Cartes P. (2008). Selenium uptake and its influence on the antioxidative system of white clover as affected by lime and phosphorus fertilization. Plant Soil.

[141] Krawczyk T. (2000). Chocolates’s hidden treasure. Inform.

[142] Clapperton J., Lockwood R., Romanczyk L., Hammerstone J. F. (1994). Contribution of genotype to cocoa (*Theobroma cacao* L.) flavour. Trop. Agric. (Trinidad).

[143] Caligiani A., Cirlini M., Palla G., Ravaglia R., Arlorio M. (2007). GC-MS detection of chiral markers in cocoa beans of different quality and geographic origin. Chirality.

[144] Azizah O., Amin I., Nawalyah a.G., Ilham A. (2007). Antioxidant capacity and phenolic content of cocoa beans. Food Chem..

[145] Rohan T.A. (1963). The precursors of chocolate aroma: application of gas chromatography in following formation during fermentation of cocoa beans. J. Food Sci..

[146] Rohan T.A., Stewart T. (1966). The precursors of chocolate aroma: production of free amino acids during fermentation of cocoa beans. J. Food Sci..

[147] Biehl B., Passern D. (1982). Proteolysis during fermentation-like incubation of cocoa seeds. J. Sci. Food Agric..

[148] Biehl B., Voigt J. Biochemistry of chocolate flavour precursors. International Cocoa Conference.

[149] Robinson T., Ranalli A.W., Phillips A.W. (1961). Changes in cocoa tannins during processing. J. Agric. Food Chem..

[150] Jinap S., Dimick P.S. (1990). Acidic characteristics of fermented dried cocoa beans from different countries of origin. J. Food Sci..

[151] Haslam E., Lilley T.H., Ho C.T., Lee C.Y., Huang M.T. (1992). Polyphenol complexation: a study in molecular recognition. Phenolic compounds in food and their effects on health.

[152] Lee C.Y., Ho C.T., Lee C.Y., Huang M.T. (1992). Enzymatic oxidation of phenolic compounds in fruits. Phenolic compounds in food and their effects on health.

[153] Bonvehi J.S., Coll F.V. (1997). Evaluation of the bitterness and astringency of polyphenolic comppunds in cocoa powder. Food Chem..

[154] Stark T., Bareuther S., Hofmann T. (2005). Sensory-guided decomposition of roasted cocoa nibs (Theobroma cacao) and structure determination of taste-active polyphenols. J. Agric. Food Chem..

[155] Pettipher G.L. (1986). Analysis of cocoa pulp and the formulation of a standardised artificial cocoa pulp medium. J. Sci. Food Agric..

[156] Wollgast J., Anklam E. (2000). Review on polyphenols in *Theobroma* cacao: Changes in composition during the manufacture of chocolate and methodology for identification and quantification. Food Res. Int..

[157] Hansen C.E., del Olmo M., Burri C. (1998). Enzyme activities in cocoa beans during fermentation. J. Sci. Food Agric..

[158] Bracco U., Grailhe N., Rostango W., Egli R. (1969). Analytical evaluation of cocoa curing in the Ivory Coast. J. Sci. Food Agric..

[159] Kyi T.M., Wan Ramli W.D., Abu Bakar M., Mohd. Wahid S., Abdul Amir H.K., Meor Zainal M.T. (2005). The kinetics of polyphenol degradation during the drying of Malaysian cocoa beans. Int. J Food Sci. Technol..

[160] De Brito E.S., Garcia N.H.P., Gallao M.I., Cortelazzo A.L., Fevereiro P.S., and Braga M.R. (2000). Structural and chemical changes in cocoa (*Theobroma cacao*) during fermentation, drying and roasting. J. Sci. Food Agric..

[161] Miller K.B., Stuart D.A., Smith N.L., Lee C.Y., Mchale N.L., Flanagan J.A., Boxin O.U., Hurst W.J. (2006). Antioxidant activity and polyphenol and procyanidin contents of selected commercially available cocoa-containing and chocolate products in the United States. J. Agric. Food Chem..

[162] Gotti R., Furlanetto S., Pinzauti S., Cavrini V. (2006). Analysis of catechins in *Theobroma cacao* beans by cyclodextrin-modified micellar electrokinetic chromatography. J. Chromatogr. Anal..

[163] Bywaters H.W. (1930). Modern methods of cocoa and chocolate manufacture. J. Franklin Inst..

[164] Gu L., House S.E., Wu X., Ou B., Prior R.L. (2006). Procyanidin and catechin contents and antioxidant capacity of cocoa and chocolate products. J. Agric. Food Chem..

[165] Cooper K.A., Campos-Gimenez C., Alvarez D.J., Rytz A., Nagy K., Williamson G. (2008). Predictive relationship between polyphenol and nonfat cocoa solids content of chocolate. J. Agric. Food Chem..

[166] Nelson B.C., Sharpless K.E. (2003). Quantification of the predominant monomeric catechins in baking chocolate standard reference material by LC/APCI-MS. J. Agric. Food Chem..

[167] Scalbert A., Williamson G. (2000). Dietary intake and bioavailability of polyphenols. J. Nutr..

[168] Donovan J.L., Crespy V., Oliveira M., Cooper K.A., Gibson B.B., Williamson G. (2006). (+)-Catechin is more bioavailable than (-)-catechin: Relevance to the bioavailability of catechin from cocoa. Free Rad. Res..

[169] Fraga C.G., Martino V.S., Ferraro G.E., Coussio J.D., Boveris A. (1987). Flavonoids as antioxidants evaluated by in vitro and in situ liver chemiluminescence. Biochem. Pharmacol..

[170] Schramm D.D., Wang J.F., Holt R.R., Ensunsa J.L., Gonsalves J.L., Lazarus S.A., Schmitz H.H., German J.B., Keen C.L. (2001). Chocolate procyanidins decrease the leukotriene-prostacyclin ratio in humans and human aortic endothelial cells. Am. J. Clin. Nutr..

[171] Holt R.R., Lazarus S.A., Sullards M.C., Zhu Q.Y., Schramm D.D., Hammerstone J.F., Fraga C.G., Schmitz H.H., Keen C.L. (2002). Procyanidin dimmer B2 [epicatechin-(4β-8)-epicatechin] in human plasma after the consumption of a flavanol-rich cocoa. Am. J. Clin. Nutr..

[172] Murphy K.J., Chronopoulos A.K., Singh I., Francis M.A., Moriarty H., Pike M.J., Turner A.H., Mann N.J., Sinclair A.J. (2003). Dietary flavanols and procyanidin oligomers from cocoa (Theobroma cacao) inhibit platelet function. Am. J. Clin. Nutr..

[173] Baba S., Osakabe N., Yasuda A., Natsume M., Takizawa T., Nakamura T., Terao J. (2000). Bioavailability of (-)-epicatechin upon intake of chocolate and cocoa in human volunteers. Free Rad. Res..

[174] Richelle M., Tavazzi I., Enslen M., Offord E. (1999). Plasma kinetics in man of epicatechin from black chocolate. Eur. J. Clin. Nutr..

[175] Baba S., Osakabe N., Natsume M., Terao J. (2002). Absorption and urinary excretion of procyanidin B2 [epicatechin-(4β-8)-epicatechin] in rats. Free Rad. Biol. Med..

[176] Depeint F., Gee J.M., Williamson G., Johnson I.T. (2002). Evidence for consistent patterns between flavonoid structures and cellular activities. Proc. Nutr. Soc..

[177] Spencer J.P., Schroeter H., Shenoy B., Srai S.K., Debnam E., Rice-Evans C. (2001). Epicatechin is the primary bioavailable form of the procyanidin dimmers B2 and B5 after transfer across the small intestine. Biochem. Biophys. Res. Comm..

[178] Da Silva E.L., Piskula M., Terao J. (1998). Enhancement of antioxidative ability of rat plasma by oral administration of epicatechin. Free Rad. Biol. Med..

[179] Natsume M., Osakabe N., Oyama M., Sasaki M., Baba S., Nakamura Y., Osawa T., Terao J. (2003). Structures of (-)-epicatechin gkucuronide identified from plasma and urine after oral ingestion of (-)-epicatechin: differences between human and rat. Free Rad. Biol. Med..

[180] Baba S., Osakabe N., Natsume M., Muto Y., Takizawa T., Terao J. (2001). Absorption and urinary excretion of (-)-epicatechin after administration of different levels of cocoa powder or (-)-epicatechin in rats. J. Agric. Food Chem..

[181] Ferruzzi M.G., Green R,J. (2006). Analysis of catechins from milk–tea beverages by enzyme assisted extraction followed by high performance liquid chromatography. Food Chem..

[182] Serafini M., Bugianesi R., Maiani G., Valtuena S., De Santis S., Crozier A. (2003). Plasma antioxidants from chocolate. Nature.

[183] Wollgast J., Anklam E. (2000). Polyphenols in chocolate: Is there a contribution to human health?. Food Res. Int..

[184] Baxter NJ, Lilley T.H., Haslam E., Williamson M.P. (1997). Multiple interactions between polyphenols and a salivary protein-rich protein result in complexation and precipitation. Biochemistry.

[185] Brunet M.J., Blade C., Salvado M.J., Arola L. (2002). Human Apo A-I and rat transferrin are the principal plasma proteins that bind wine catechins. J. Agric. Food Chem..

[186] Zhu Y.Z., Hammerstone J.F., Lazarus S.A., Schmitz H.H., Keen C.L. (2003). Stabilizing effect of ascorbic acid on flavan-3-ols and dimeric procyanidins from cocoa. J. Agric. Food Chem..

[187] Klimczak I., Malecka M., Szlachta M., Gliszczynska-Swiglo A. (2007). Effect of storage on the content of polyphenols, vitamin C and the antioxidant activity of orange juices. J. Food Compos. Anal..

[188] Zhu Q.Y., Holt R.R., Lazarus S.A., Ensunsa J.L., Hammerstone J.F., Schmitz H.H., Keen C.L. (2002). Stability of the flavan-3-ols epicatechin and catechin and related dimeric procyanidins derived from cocoa. J. Agric. Food Chem..

[189] Deprez S., Brezillon C., Rabot S., Philippe C., Mila I., Lapierre C., Scalbert A. (2000). Polymeric proanthocyanidins are catabolized by human colonic microflora into low-molecular-weight phenolic acids. J. Nutr..

[190] Spencer J.P.E., Chaudry F., Pannala A.S., Srai S.K., Debnam E, Rice-Evans C. (2000). Decomposition of cocoa procyanidins in the gastric milieu. Biochem. Biophys. Res. Comm..

[191] Silberberg M., Gil-Izquierdo A., Combaret L., Remesy C., Scalbert A., Morand C. (2006). Flavanone metabolism in healthy and tumor-bearing rats. Biomed. Pharmacother..

[192] Kumazawa T., Seno H., Lee X-P., Ishii A., Watanabe-Suzuki K., Sato K., Suzuki O. (1999). Extraction of methylxanthines from human body fluids by solid-phase microextraction. Anal. Chim. Acta.

[193] Lelo A., Miners J.O., Robson R., Birkett D.J. (1986). Assessment of caffeine exposure: caffeine content of beverages, caffeine intake, and plasma concentrations of methylxanthines. Clin. Pharmacol. Ther..

[194] Lelo A., Birkett D.J., Robson R.A., Miners J.O. (1986). Comparative pharmacokinetics of caffeine and its primary demethylated metabolites paraxanthine, theobromine and theophylline in man. Brit. J. Clin. Pharmacol..

